# Gender differences in peer review outcomes and manuscript impact at six journals of ecology and evolution

**DOI:** 10.1002/ece3.4993

**Published:** 2019-03-04

**Authors:** Charles W. Fox, C. E. Timothy Paine

**Affiliations:** ^1^ Department of Entomology University of Kentucky Lexington Kentucky; ^2^ Ecosystem Management, School of Environmental and Rural Science University of New England Armidale New South Wales Australia

**Keywords:** bias, citations, discrimination, gender, peer review, scholarly publishing

## Abstract

The productivity and performance of men is generally rated more highly than that of women in controlled experiments, suggesting conscious or unconscious gender biases in assessment. The degree to which editors and reviewers of scholarly journals exhibit gender biases that influence outcomes of the peer‐review process remains uncertain due to substantial variation among studies. We test whether gender predicts the outcomes of editorial and peer review for >23,000 research manuscripts submitted to six journals in ecology and evolution from 2010 to 2015. Papers with female and male first authors were equally likely to be sent for peer review. However, papers with female first authors obtained, on average, slightly worse peer‐review scores and were more likely to be rejected after peer review, though the difference varied among journals. These gender differences appear to be partly due to differences in authorial roles. Papers for the which the first author deferred corresponding authorship to a coauthor (which women do more often than men) obtained significantly worse peer‐review scores and were less likely to get positive editorial decisions. Gender differences in corresponding authorship explained some of the gender differences in peer‐review scores and positive editorial decisions. In contrast to these observations on submitted manuscripts, gender differences in peer‐review outcomes were observed in a survey of >12,000 published manuscripts; women reported similar rates of rejection (from a prior journal) before eventual publication. After publication, papers with female authors were cited less often than those with male authors, though the differences are very small (~2%). Our data do not allow us to test hypotheses about mechanisms underlying the gender discrepancies we observed, but strongly support the conclusion that papers authored by women have lower acceptance rates and are less well cited than are papers authored by men in ecology.

## 1 INTRODUCTION

Review of manuscripts by peers has been a key feature of scholarly publishing for nearly three centuries (Spier, 2002). Peer review improves the quality of manuscripts before they are published (Bakanic, McPhail, & Simon, 1987; Goodman, Berlin, Fletcher, & Fletcher, 1994) and helps editors identify contributions that will be the most impactful (Li & Agha, 2015; Paine & Fox, 2018). However, peer review may also be subject to systemic biases that influence editorial outcomes (Lee, Sugimoto, Zhang, & Cronin, 2013). For example, reviewers rate papers with famous authors, or authors from prestigious institutions, more highly (Tomkins, Zhang, & Heavlin, 2017). Editors and reviewers may also exhibit biases, conscious, or unconscious, against authors who speak a different language or reside in a different country from themselves (Lee et al., 2013; Murray et al., 2018). However, gender bias, specifically bias against female authors, has garnered the most attention.

A wide diversity of research demonstrates that the productivity and performance of men is generally rated higher than that of women, even in controlled experiments (Moss‐Racusin, Dovidio, Brescoll, Graham, & Handelsman, 2012, and references therein). However, the frequency and degree to which peer review of scholarly manuscripts and grant proposals discriminates against women remains a subject of significant debate. Some experimental studies, in which author genders are manipulated (e.g., investigators manipulate the gender of names on author bylines), have found that papers with male‐sounding author names are rated more highly than those with female‐sounding names (Knobloch‐Westerwick, Glynn, & Huge, 2013; Krawczyk & Smyk, 2016), though there are exceptions (Borsuk, Budden, Leimu, Aarssen, & Lortie, 2009). In contrast, correlational studies of manuscript or grant review commonly find it to be gender neutral (e.g., no discrepancy in outcomes between papers with male vs. female authors; Buckley, Sciligo, Adair, Case, & Monks, 2014; Edwards, Schroeder, & Dugdale, 2018; Fox, Burns, Muncy, & Meyer, 2016; Heckenberg & Druml, 2010; Lane & Linden, 2009; Primack, Ellwood, Miller‐Rushing, Marrs, & Mulligan, 2009 for manuscript review; Cañibano, Otamendi, & Andújar, 2009; Leemann & Stutz, 2008; Ley & Hamilton, 2008; Marsh, Jayasinghe, & Bond, 2008; Marsh, Jayasinghe, & Bond, 2011; Mutz, Bornmann, & Daniel, 2015; Reinhart, 2009; Sandström & Hällsten, 2008, and references therein, for grant reviews), though there are exceptions in which men (Kaatz et al., 2016; Ledin, Bornmann, Gannon, & Wallon, 2007; Murray et al., 2018; Walker, Barros, Conejo, Neumann, & Telefont, 2015) or women (Lerback & Hanson, 2017) have higher success rates. In some studies, gender differences are detected in some but not all stages of the manuscript or grant review process (e.g., Handley et al., 2015; Van den Besselaar & Leydesdorff, 2009). The meta‐analysis of Bornmann, Mutz, and Daniel (2007) found that men are overall more likely to receive grants than are women (though effect size was small), whereas the meta‐analysis of Marsh, Bornmann, Mutz, Daniel, and O'Mara (2009) found no evidence that men have higher grant success than women, and presented some evidence that women have higher success than men after controlling for critical explanatory variables. Experiments blinding editors or reviewers to author identity also generally find no effect on gender differences in outcomes (Blank, 1991; Carlsson, Löfgren, & Sterner, 2012; Ross et al., 2006).

The studies testing for gender inequalities in peer review (even the meta‐analyses aggregating these studies) are thus highly variable in results and conclusions. There are a variety of possible explanations for this variation. Studies vary in their research subjects; for example, peer reviewers for academic journals and granting agencies are generally professional scientists, whereas the manipulative studies that detect gender effects on assessment scores often use students as the evaluators, possibly contributing to the differences observed between correlative and manipulative studies. Also, gender differences may be obscured in correlational studies by other biases (such as prestige bias) and by the wide variation in quality and significance of the documents being assessed. It is notable that, although few correlational studies detect statistically significant effects of gender on peer review, effect sizes are usually in the hypothesized direction (bias against women). Regardless of the reason why there is so little consistency of conclusions among studies, it leaves unresolved questions about the frequency and magnitude of gender differences in the outcomes of scholarly peer review.

Differences in the assessment of the quality and significance of scientific contributions can continue after an article has been published. For example, when ecologists were asked to propose lists of papers that ecology students should read before completing their dissertation, the proposed lists were dominated by male‐authored papers, though female ecologists proposed papers that included, on average, more female authors (Bradshaw & Courchamp, 2018). In ecology, men also accumulate more citations (across their entire portfolio of papers) than do women, and men have, on average, higher H‐indices (Cameron, White, & Gray, 2016). However, that study found no evidence that citations *per paper* differed between men and women in ecology, consistent with two earlier studies of the ecology literature that failed to find gender differences in citations (Borsuk et al., 2009; Leimu & Koricheva, 2005). These results contrast with numerous studies in other disciplines, most of which show that papers authored by women are less well cited than papers authored by men, for example, across all sciences (Bendels, Müller, Brueggmann, & Groneberg, 2018; Larivière, Ni, Gingras, Cronin, & Sugimoto, 2013; Sugimoto, Lariviere, Ni, Gingras, & Cronin, 2013), in the social sciences (Carter, Smith, & Osteen, 2017; Dion, Sumner, & Mitchell, 2018), and in a variety of other disciplines (Tahamtan, Afshar, & Ahamdzadeh, 2016), though exceptions exist. Thus, as with studies of peer review, studies examining manuscript impact vary substantially in their conclusions.

The goals of this paper were to test whether author gender predicts (a) the outcomes of editorial and peer review or (b) the number of citations that papers obtain postpublication in journals of ecology. To test for relationships between author gender and outcomes of peer review, we examine two datasets. First, we test for relationships between author gender and editorial and peer‐review outcomes in a dataset that contains detailed information on authors and the peer‐review process experienced by >23,000 research manuscripts submitted to six journals in ecology and evolution between 2010 and 2015. Our dataset is large enough to have the statistical power necessary to detect small but meaningful gender differences in outcomes. Second, we survey authors of published manuscripts to obtain the submission histories of those manuscripts (>12,000 responses), including whether their papers had been rejected from at least one prior journal before being accepted at the journal that eventually published the paper. To test for relationships between author gender and citations, we analyze author and citation data extracted from Clarivate Analytics Web of Science (WoS) for all journal articles published between 2009 and 2015 in journals categorized in the domain of ecology.

## 2 METHODS

### 2.1 Datasets

#### 2.1.1 Submission dataset

We extracted all metadata and peer‐review details for all manuscripts submitted to six ecology and evolution journals from *ScholarOne Manuscripts*. We included manuscripts submitted between 1 January 2010 and 30 June 2015 for *Functional Ecology*, *J Animal Ecology*, *J Applied Ecology*, *J Ecology*, and *Methods in Ecology and Evolution* (this journal received its first ever submission on 13 August 2009), and between 1 January 2010 and 31 December 2015 for *Evolution*. The dataset includes only standard research papers (called a “Research Article” at *Methods in Ecol Evol*, an “Original Article” at *Evolution*, and a “Standard Paper” at the other journals). We consider only the first submission of a manuscript; revisions and resubmissions were excluded (so that we do not double count papers). Data in ScholarOne are author‐entered and so author lists in the database are sometimes incomplete and often incorrectly ordered. We thus determined authorship order and corresponding authorship on papers from the cover page of the submitted manuscript. The dataset includes 23,713 manuscripts, 22,592 of which have more than one author, and 1,121 of which have a single author. A more detailed evaluation of the gender distribution of authorships in this dataset is presented in Fox, Ritchey, and Paine (2018).

Author gender was determined using the online database http://genderize.io. This database includes >200,000 distinct names and assigns a probability that each name is male or female given the distribution of genders for these names in the database. If an author's name was not listed in genderize.io, or was listed but had less than a 95% probability of being one gender, we used an Internet search to determine gender. To do so, we searched for individual web pages or entries in online databases that included a photograph of the individual or other information suggesting their gender. In the dataset of submitted papers, we were able to genderize ~98% of all authors (98.4% of first authors and 98.0% of last authors).

Throughout our analysis, we distinguish two stages of the editorial and peer‐review process. First, papers are screened by senior and/or Associate/Handling Editor editors before being sent for review; a large proportion of papers are declined at this stage (Fox & Burns, 2015). Once reviews are obtained, a final decision is made on the paper (the paper is either declined or is invited for revision/resubmission). The six journals examined here differ in the frequency with which they invite revision (minor or major) versus reject papers with the option to resubmit (often used in place of inviting a major revision). We thus performed two analogous sets of analyses, one in which we consider an invitation to revise as the only positive outcome and the other in which we consider both an invitation to revise and an invitation to resubmit (reject with resubmission invited) as positive outcomes. See the section “Editorial decisions after review,” below, for details.

#### 2.1.2 Author survey

We obtained metadata for all articles published between 2009 and 2015 in 146 journals classified by Clarivate Analytics Web of Science (WoS) in the research domain of Ecology. Review and methods journals such as the *Trends and Annual Reviews* series and *Methods in Ecology & Evolution* were excluded. We sent questionnaires to the corresponding authors of a subsample of these manuscripts, including only one randomly selected paper per corresponding author. Further details about the sampling scheme and dataset are presented in Paine and Fox (2018).

Using the Qualtrics platform, we sent questionnaires to each corresponding author to request information about the publication history of their paper. The complete questionnaire is presented in Paine and Fox (2018). Important for our analyses here is that we requested details on the history of the published article, including the journals to which the manuscript had previously been submitted, whether it was an invited manuscript, and the year and outcome of each submission.

In total, 52,543 unique corresponding authors were surveyed. A total of 12,655 authors, 24.1% of those contacted, responded to our questionnaire. After removal of incomplete or unintelligible responses and invited papers, and journals to which fewer than 100 manuscripts were available, we have histories for 8,597 manuscripts from 81 journals.

Author names were genderized using the same process as detailed above. We genderized 93.7% of first authors and 95.1% of last authors.

#### 2.1.3 Citations to papers authored by men versus women

To test for the relationships between author gender and the number of citations obtained by manuscripts, we extracted metadata from Clarivate Analytics Web of Science for all manuscripts published from 2009 to 2015 in the ecology domain. We excluded review journals such as *Trends in Ecology and Evolution* and the *Annual Review of Ecology, Evolution, and Systematics*, as most papers in those journals are invited. We also excluded journals with fewer than 100 papers, and excluded review papers, commentaries, perspectives, editorials, brief communications, and other types of papers not considered typical full‐length research studies. This dataset includes 108,295 studies published in 142 journals.

We used journal impact factors obtained from *Clarivate* *Analytics *Journal Citation Reports. Because manuscripts are typically submitted to a journal one or two years before their eventual publication, we used journal impact factors for annual period that was two years prior to the publication year of the focal manuscript as our measure of journal rank at the time of manuscript submission. These impact factors are typically made public half‐way through the following year and thus would be the most recently available impact factors an author could consider when submitting their manuscript. Impact factors were log‐transformed to reduce heteroscedasticity.

Author names were genderized using the same process detailed above. We genderized 93.7% of authors (93.7% of first authors and 95.2% of last authors).

### 2.2 Analyses

For papers with more than one author, our analyses of author gender focus on the first, last, and corresponding author, rather than overall author gender ratio. This is because middle authors generally have less prominent roles in manuscript preparation and submission and, in our dataset, the corresponding author was either the first author or the last author for 95.1% of papers for which a corresponding author was identified. In the appendix, we present analyses for overall author gender ratio (the proportion women), analogous to those presented in the main text for first, last, and corresponding author gender; the main outcomes of those analyses are the same as those for first and last author, with minor differences only in the details. Few papers had a single author, comprising only 4.7% of the submitted papers dataset (6.4% of the questionnaire dataset and 5.8% of the WoS dataset); these papers are analyzed separately from multiauthored papers.

Throughout our presentation, we identify the last author as the “senior author.” This is because ecologists tend to assume that the last author is the senior researcher (e.g., head of laboratory) under whose guidance the research was executed (Duffy, 2017).

#### 2.2.1 Submitted manuscript dataset

Each manuscript represents a single data point that includes one first author, one senior author, one corresponding author, and one author gender ratio. Thus, all analyses are performed considering individual manuscripts as independent data points. To test for gender differences in editorial outcomes (a binomial outcome), we used logistic regression (SAS Proc Logistic) to model *PositiveOutcome*[yes/no] = Journal + *Year* + *AuthorGender*[female/male] + 2*‐way interactions*, with all independent variables as fixed effects (*Journal* is included as a fixed effect to allow testing for interactions, e.g., variation in gender effects among journals). Review scores are not binomial, and vary substantially among journals and years, dependent on the journal rating scales and rating guidelines (which vary among years within journals). We thus first standardized review scores (average score across reviews within each paper) to a mean of 0 and variance of 1.0 within each journal‐year combination. *ReviewScore *therefore has units of standard deviations and measures the deviation from the overall average review score of 0, with *higher* scores being better. We then tested for gender effects of peer‐review scores using analysis of variance (SAS Proc GLM), modeling *ReviewScore* = *Journal* + *AuthorGender* + *Journal* *‐x‐AuthorGender interaction* (*Journal* is included to allow testing for the interaction; *Year* is not included because all year‐journal combinations are defined to have the same mean).

For presentation in figures, we calculated a female:male success ratio, which is the probability of a positive outcome when the author is female divided by the probability of a positive outcome when the author is male, for which a ratio of 1.0 indicates that there is no gender difference in manuscript outcomes. Means of this success ratio were calculated for each journal‐year combination, then averaged across years within journals. All standard errors for manuscript outcomes and the success ratio are calculated from the among‐year variance within journals (for averages within journals) or from the among‐journal variance (for overall means across journals).

Least‐squares means, where presented, are calculated using the LSMeans statement in either SAS Proc Logistic (for binomial variables; probabilities calculated using the ILINK switch), or SAS Proc GLM.

#### 2.2.2 Author questionnaire dataset

To assess the effect of author gender on the probability of manuscript rejection across the 81 journals in the questionnaire dataset, we predicted the probability of a positive outcome as a binomial response given *AuthorGender*. We allowed *AuthorGender* to interact with the logarithm of the journal impact factor (*JIF*), to assess whether gender bias varied with journal prominence. Journal was included as a random intercept, to account for variation in rejection rates among journals independent of JIF. Thus, the generalized mixed‐effect model had the form *PositiveOutcome*[yes/no] = *AuthorGender* * log(*JIF)* + (1|*Journal*) and was fit using the lme4 library in R 3.5.1. The effect of the gender of the first and last author on *PositiveOutcome* did not vary significantly with *JIF *(*p* ≥ 0.74). Therefore, we dropped these interactions from the models.

#### 2.2.3 Published paper dataset

To assess the effect of author gender on the number of citations obtained by articles published in the 142 journals in the Web of Science dataset, we predicted the number of citations given *AuthorGender *using a Poisson error distribution. We allowed *AuthorGender* to interact with the logarithm of the impact factor of the journal (*JIF*), to assess whether gender bias varied with journal prominence. The year of publication was included as a random intercept, to account for the nonlinear accumulation of citations through time. Thus, the generalized mixed‐effect model had the form *NumberofCitations* = *AuthorGender* * log(*JIF)* + (1|*Year*) and was fit using the lme4 library in R 3.5.1. For single‐authored papers, the effect of gender on *NumberofCitations* did not vary significantly with *JIF *(*p* = 0.099). Therefore, we dropped this interaction from the model.

### 2.3 Permits and permissions

The datasets analyzed here include personal identifiers. Thus, it was essential to maintain the confidentiality of all participants. The datasets provided online (Dryad) were thus anonymized to maintain the privacy of authors. CW Fox was the only person to have access to nonanonymized versions of the submitted papers dataset (University of Kentucky Institutional Review Board approval, IRB 15–0890), and CET Paine was the only person with access to nonanonymized survey data. Human subject ethical approval for the questionnaire aspects of this study was obtained from the University of Stirling.

## 3 RESULTS

### 3.1 Gender differences in outcomes of the editorial and peer‐review process

#### 3.1.1 Single‐authored papers

We found no evidence that single‐author papers submitted by women were less likely to be sent for peer review (proportion reviewed = 40.2 ± 7.5% for women vs. 43.5 ± 5.8% for men; $\chi_{1}^{2}$ = 0.56, *p* = 0.45), obtained lower review scores when reviewed (review scores standardized to the entire dataset = −0.07 ± 0.17 vs. −0.08 ± 0.05; *F*_1,472_ = 0.10, *p* = 0.75), or were less likely to be invited for revision (30.6 ± 6.7 vs. 27.7 ± 1.9%; $\chi_{1}^{2}$ = 0.18, *p* = 0.67) or revision +resubmission (47.1 ± 6.3 vs. 48.2 ± 5.9%; $\chi_{1}^{2}$ = 0.90, *p* = 0.34) if reviewed. Thus, cumulative throughout the entire process, we found no evidence that single‐authored papers submitted by women were less likely to have positive outcomes, whether we consider a positive outcomes as just being invited to submit a revision (12.5 ± 2.4 vs. 11.7 ± 1.6%; $\chi_{1}^{2}$ = 0.90, *p* = 0.34) or being invited for revision or resubmission (20.0 ± 5.9 vs. 21.5 ± 5.2%; $\chi_{1}^{2}$ = 0.46, *p* = 0.50; means ± SEMs are calculated by first averaging across years within journals, then across journals).

#### 3.1.2 Multiauthor papers

##### Desk rejection

**Figure 1:**
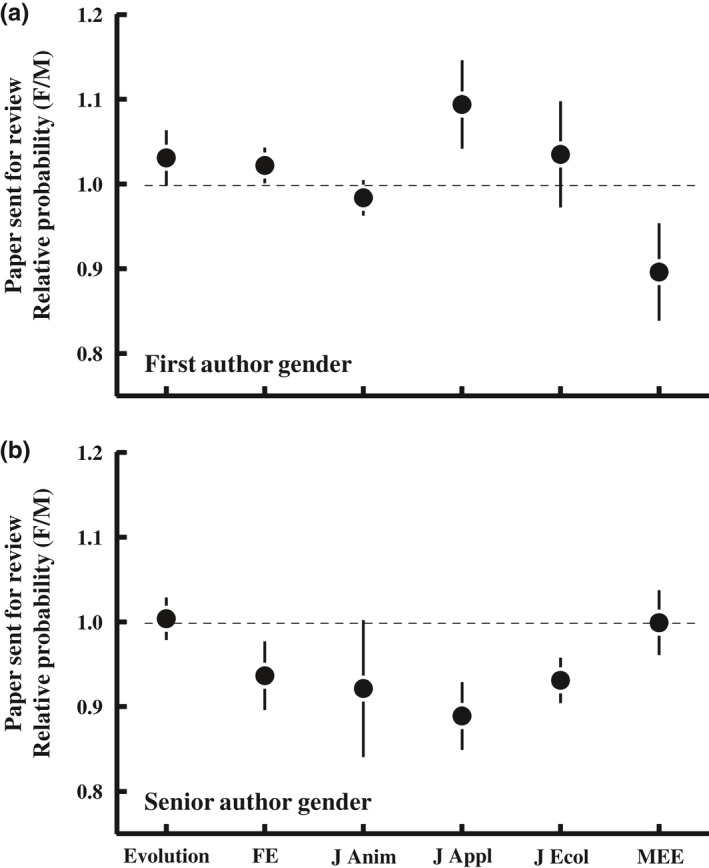
Papers with male and female first authors were equally likely to be sent for peer review, whereas papers with female senior authors were slightly but significantly less likely to be sent for peer review, at six journals of ecology and evolution. Values >1 indicate that papers with female authors are more likely to be sent for peer review, whereas values <1 indicate that papers with female authors are less likely to be sent for peer review. The mean proportions of papers sent for review for each journal‐gender combination are in Figure A1. Logistic regression (SAS Proc Logistic): *PaperReviewed*[yes/no] = *Journal* + *Year *+ *AuthorGender* + *2‐way interactions*. (a) First author gender: $\chi_{1}^{2}$ = 0.25, *p* = 0.62; (b) Senior author gender: $\chi_{1}^{2}$ = 7.35, *p* = 0.007.

**Figure A1:**
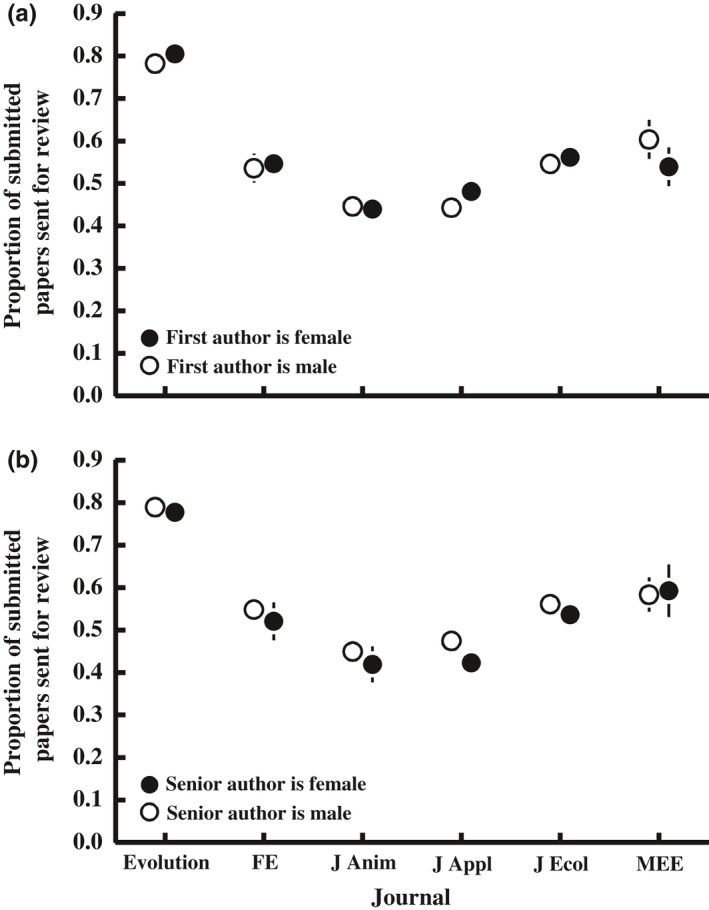
The proportion of submitted papers that are sent for peer review at six journals of ecology and evolution varies among journals. Analyses as in Figure 1. Means are calculated by averaging across yearly means within journals (±*SEM*). Error bars are often smaller than the points

The proportion of papers sent for peer review varied substantially among journals (Figure 1) and across years. On average across journals, papers with female versus male first authors were equally likely to be sent for peer review (Figure 1a): 56.2 ± 5.2% of papers with female first authors were sent for review and 56.0 ± 5.1% of papers with male first authors were sent for review (average of journal means). This varied among journals (significant interaction; Figures 1 and A1A1); however, the success ratio ranged from a low of 0.90 to a high of 1.09, with an average of 1.01 (Figure 1a).

Papers with female senior authors were slightly, but significantly, less likely to be sent for peer review compared to papers with male senior authors: 54.5 ± 5.4% of papers with female senior authors were sent for review, compared to 56.8 ± 4.9% for male authors (Figure 1b). On average, papers with female last authors were 95 ± 1% as likely to be sent for review as were papers with male last authors (ratio of success of female:male papers = 0.95). As was observed for first author gender, the difference between male and female senior authors in the proportion of their papers sent for review varied among journals, although the pattern differed from that for first author gender (e.g., the direction of the gender difference for *J Appl* switched directions, from females>males to females <males.

We found no evidence that gender of the corresponding author influenced the likelihood that a paper would be sent for peer review (logistic regression, statistical model as in Figure 1; $\chi_{1}^{2}$ = 2.54, *p* = 0.11); 56.7 ± 5.2% of papers authored by women were sent for peer review, and 55.6 ± 5.1% of papers authored by men were sent for peer review (averaged across years within journals, then across journals within years), a ratio of female:male success of 1.02.

##### Review scores

**Figure 2:**
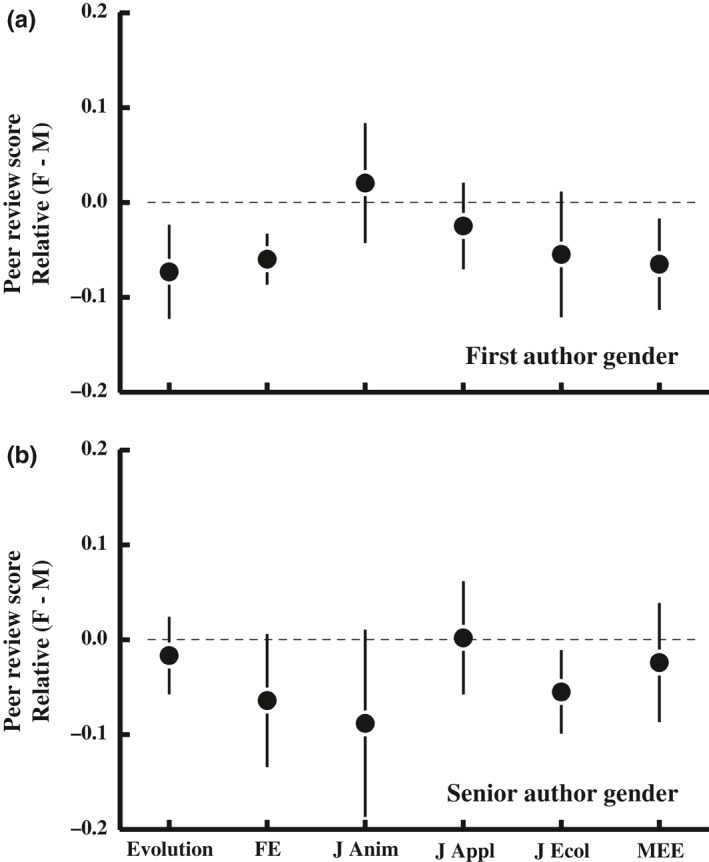
Papers with female first authors obtained slightly but significantly lower review scores than did papers with male first authors, though there was no difference in the scores received by male and female senior authors. Review scores were standardized to a mean of 0 and a standard deviation of 1.0 within journals and years so that all journals are on the same scale. Values >0 indicate that papers with female authors obtain higher average review scores, whereas values <1 indicate that papers with female authors obtain lower average review scores. The mean review scores for each journal‐gender combination are in Figure A2. Analysis of variance: *ReviewScore* = *Journal* + *AuthorGender* + interaction. Because scores were standardized within each journal‐year combination, journal is only included to test for journal*gender interactions. First author gender: *F*_1,11667_ = 6.50, *p* = 0.01; Senior author gender: *F*_1,11650_ = 2.54, *p* = 0.11.

Of papers sent for review, papers with female first authors obtained slightly but significantly lower review scores than did papers with male first authors (Figure 2a). Though this difference is statistically significant, it is very small, averaging (across journals) just 0.04 ± 0.02 standard deviations. No difference in review scores was observed for female versus male senior authors (Figure 2b), but papers with a female corresponding author obtained significantly lower review scores (−0.04 ± 0.01 vs. 0.01 ± 0.01, units =standard deviations; *F*_1,11583_ = 10.65, *p* = 0.001; analysis of variance, model as in Figure 2).

##### Editorial decisions after review

**Figure 3:**
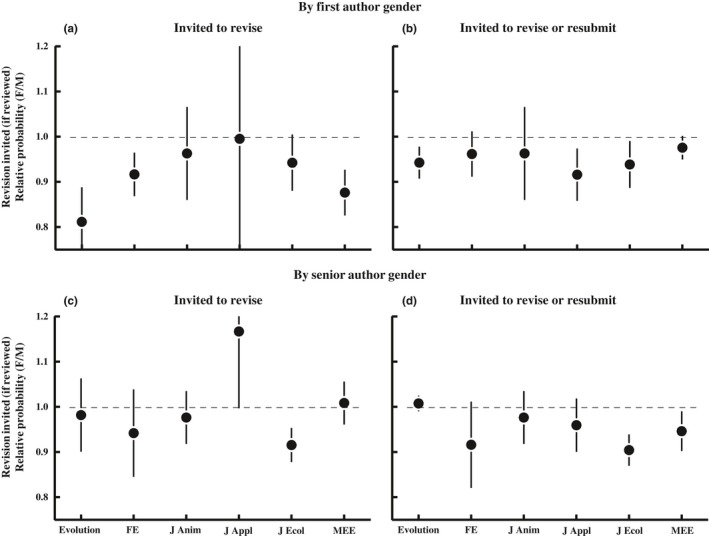
The relative proportion of *reviewed papers* that had positive final outcomes for papers with female versus male authors. Values >1 indicate that papers with female authors are more likely to have positive outcomes, whereas values <1 indicate that papers with female authors are less likely to have positive outcomes. The left panels count as a positive outcome‐only papers that were invited for minor or major revision. The right panels include papers that were invited for minor or major revision or were rejected but invited to resubmit. Three of the journals (*Evolution*, *J Applied Ecology,* and *Methods in Ecology and Evolution*) make frequent use of “reject with resubmission invited,” but three others use this decision category rarely. The mean proportion of papers with positive outcomes for each journal‐gender combination are in Figure A3. Logistic regression: *PositiveOutcome*[yes/no] = *Journal* + *Year *+ *AuthorGender* + *2‐way interactions*. Revision invited by first author gender (Panel a): $\chi_{1}^{2}$ = 15.4, *p* < 0.001; revision or resubmission invited by first author gender (Panel b): $\chi_{1}^{2}$ = 7.72, *p* = 0.006; revision invited by senior author gender (Panel c): $\chi_{1}^{2}$ = 0.18, *p* = 0.67; revision or resubmission invited by senior author gender (Panel d): $\chi_{1}^{2}$ = 4.42, *p* = 0.04.

**Figure 4:**
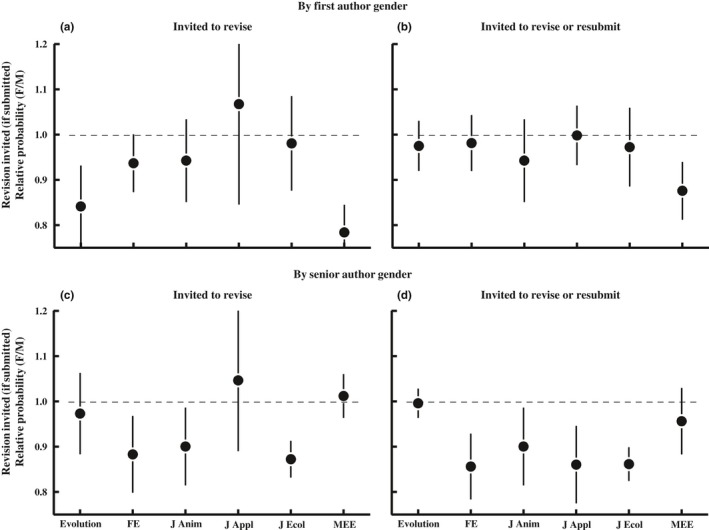
The proportion of *all submitted papers* that had positive final outcomes, cumulative through the entire pre‐ and postreview process. This figure differs from Figure 8 in that negative outcomes include both papers that were declined without review (desk rejections) and those declined after review. Values >1 indicate that papers with female authors are more likely to have positive outcomes, whereas values <1 indicate that papers with female authors are less likely to have positive outcomes. The left panels count as a positive outcome‐only papers that were invited for minor or major revision. The right panels include papers that were invited either for revision or were rejected but invited to resubmit. The mean proportion of papers with positive outcomes for each journal‐gender combination are in Figure 4. Logistic regression: *PositiveOutcome*[yes/no] = *Journal* + *Year *+ *AuthorGender* + *2‐way interactions*. Revision invited by first author gender (Panel a): $\chi_{1}^{2}$ = 12.9, *p* < 0.001; revision or resubmission invited by first author gender (Panel b): $\chi_{1}^{2}$ = 6.54, *p* = 0.01; revision invited by senior author gender (Panel c): $\chi_{1}^{2}$ = 2.37, *p* = 0.12; revision or resubmission invited by senior author gender (Panel d): $\chi_{1}^{2}$ = 10.8, *p* = 0.001.

Once editors have reviews in hand, they must decide whether to invite revision or decline the manuscript. The six journals differ in the types of decisions they make. *FE*, *J Anim* and *J Ecol* primarily either invite revision (minor or major) or reject; they rarely offer the option to resubmit a rejected manuscript. The other journals commonly invite resubmission of manuscripts that are rejected (reject with resubmission invited). We thus perform two analogous sets of analyses. In the first, we treat a revision invitation as the only positive outcomes and treat all rejections (whether resubmission is invited or not) as a negative outcome. In the other, we consider rejections for which resubmission was invited to also be a positive outcome. These two sets of analyses are labeled as “Invited to revise” versus “Invited to revise or resubmit,” respectively, in Figures 3 and 4. The two analyses differ little for *FE*, *J Anim* and *J Ecol*, but can differ more substantially for the other three journals. Figure 3 presents relative outcomes (female:male) considering only papers sent for review, and Figure 4 presents relative outcomes for all submitted papers (cumulative through the entire process from submission to editorial decision).

*First authors—*Of papers that were sent to review, papers with female first authors were slightly less likely to be invited for revision (major or minor; Figure 3a); papers with female first authors were invited for revision 29.8 ± 3.1% of the time, compared to 32.9 ± 3.1% of the time for papers with male first authors (averaged across journals), a female: male success ratio of 0.91 (Figure 3a). However, this first author gender difference varied among journals, with the success ratio varying from a low of 0.81 to a high of 1.00 (each point in the figure is an average across years within journals). The overall difference in the success rate for female versus male first authors remained significant, but of smaller magnitude, when considering invited resubmissions as a positive outcome (Figure 3b); 48.4 ± 5.2 versus 50.9 ± 5.1% of reviewed papers were invited to revise or resubmit when first authors were female versus male (female:male success ratio = 0.95). This again varied among journals, with the female:male success ratio ranging from a low of 0.92 to a high of 0.98. The reason that the magnitude of the gender difference declined when considering resubmissions as a positive outcome is that papers authored by women were more likely to be rejected with an invitation to resubmit than were papers authored by men ($\chi_{1}^{2}$ = 7.4, *p* = 0.007; this analysis considers only reviewed papers at the three journals that make frequent use of the “reject with resubmission invited” decision).

Cumulative across the whole process (including both prereview and postreview rejections), papers with female first authors were slightly but significantly less likely to be invited for revision (Figure 4a); papers with female first authors were invited for revision 16.4 ± 1.9% of the time, compared to 18.3 ± 2.4% of the time for papers with male first authors (averaged across journals). The relative success of papers authored by female relative to male authors (average of the six journal‐specific success ratios) was 0.93 for all submitted manuscripts. This gender difference in outcomes remained significant when including resubmissions as a positive outcome (Figure 4b), though the effect size was slightly smaller, 27.4 ± 4.7 versus 29.0 ± 5.1% for female and male first authors, respectively; the female‐to‐male success ratio, averaged across journals, was 0.96.

*Senior authors*—In contrast to the significant differences in the success rate of papers with female versus male first authors, there was no overall significant gender difference in success rate of papers that were sent for review for papers with female versus male senior authors, regardless of whether we consider a positive outcome to include just a revision invitation (Figure 3c; average female:male ratio across journals = 0.95) or to also include a resubmission invitation (Figure 3d; average female:male success ratio = 0.96). There was also no gender difference in the probability a paper was invited for resubmission ($\chi_{1}^{2}$ = 1.94, *p* = 0.16). Cumulative through the entire process (considering both prereview and postreview rejects), there was no significant gender difference in success of senior authors when considering only revisions as a positive outcome (Figure 4c; female:male ratio = 0.95), but we do detect a significant difference when considering both revision and resubmission invitations as positive outcomes (Figure 4d; average female:male success ratio = 0.91).

*Corresponding authors*—Of reviewed papers, those with female corresponding authors were less likely to have a positive final outcome, whether we consider being invited to submit a revision (29.3% vs. 32.9% for men and women, respectively, female:male success ratio =0.89)($\chi_{1}^{2}$ = 16.9, *p* < 0.001) or being invited to either revise or resubmit following rejection (48.2% vs. 50.9%; success ratio = 0.95; $\chi_{1}^{2}$ = 8.2, *p* = 0.004) as positive outcomes. This gender difference in the probability that a manuscript has a positive outcome is at least partly explainable by peer‐review scores; after accounting for the gender difference in peer‐review scores (adding peer review score as a covariate in the statistical model), the gender difference in the probability of a positive outcome disappeared when positive outcomes included the invitation to revise or resubmit (female:male success ratio = 0.98; $\chi_{1}^{2}$ = 0.49, *p* = 0.48), but remained significant when positive outcomes included only the invitation to revise (success ratio = 0.89; $\chi_{1}^{2}$ = 4.22, *p* = 0.04).

Cumulative through the entire peer‐review process (both pre‐ and postreview editorial decisions), submissions with female corresponding authors were only 90% as likely to be invited for revision (16.4% vs. 18.2%; success ratio =0.90; $\chi_{1}^{2}$ = 10.5, *p* = 0.001), but 97% as likely to be invited revise or resubmit (27.7% vs. 28.7%; success ratio = 0.97; $\chi_{1}^{2}$ = 3.57, *p* = 0.06), relative to papers with male corresponding authors.

##### Do review scores account for observed differences in editorial decisions?

In the previous section, we observed that papers with female first authors were less likely to be invited for revision or resubmission after review than were papers with male first authors. Though the pattern varied among journals, and effect sizes were often quite small, it is notable that the direction of the difference was nearly always the same, with female‐authored papers less likely to obtain a positive outcome after peer review. However, we also observed that papers with female first authors commonly received lower (albeit only slightly) peer‐review scores. We thus ask whether this difference in peer‐review scores, which are known to be the major variable affecting editorial decisions after review (Fox et al., 2016), can account for the gender difference in editorial decisions for papers that have been reviewed.

When including peer‐review scores (the data in Figure 2) as a covariate in the statistical models testing for gender differences in editorial decisions (those in Figure 3), we find that peer‐review score is the overwhelming major predictor of editorial decisions for reviewed papers whether you consider just an invitation to revise ($\chi_{1}^{2}$ > 2,613, *p* < 0.001) or both an invitation to revise or resubmit ($\chi_{1}^{2}$ > 2,803, *p* < 0.001) as positive outcomes. Notably, the differences in success rates for papers female versus male first authors became nonsignificant when you consider resubmissions to be a positive outcome (first author: $\chi_{1}^{2}$ = 1.06, *p* = 0.30; female:male success ratio = 0.97), but remained significant when only an invitation to revise was considered a positive outcome ($\chi_{1}^{2}$ = 5.28, *p* = 0.002; success ratio = 0.89). The effect of senior author gender on manuscript outcomes remained nonsignificant after including peer review scores in the model whether we considered resubmission invitations to be a positive outcome ($\chi_{1}^{2}$ = 1.87, *p* = 0.17; success ratio = 0.98) or not ($\chi_{1}^{2}$ = 0.21, *p* = 0.65; success ratio = 1.09).

##### Corresponding authorship and editorial decisions

In a previous paper, Fox et al. (2018) found that ~20% of first authors defer corresponding authorship to one of their coauthors, and that female first authors defer corresponding authorship more often than do male first authors. The corresponding author listed on the cover page of the manuscript is the author that submitted the paper to the journal for >99% of papers considered by *Functional Ecology* (Fox, Burns, Muncy, & Meyer, 2017). We thus asked whether deferring corresponding authorship was predictive of how well a submitted manuscript fares after submission, and whether the gender difference in corresponding authorship could account for the gender differences in peer‐review outcomes observed in this study. We do this by adding *FirstIsCorrespondingAuthor *[yes/no], plus two‐way interactions containing this term, to the statistical models testing for first author gender effects in Figures 1, 2, 3, 4.

**Figure 5:**
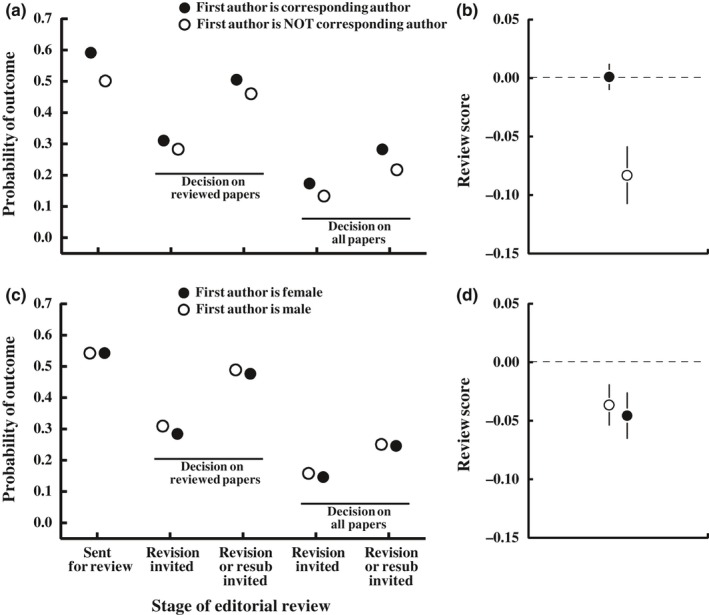
Papers for which the first author served as corresponding author fare much better throughout the peer‐review process (a, b). Means ± standard errors are from the statistical models below (LSMeans). Accounting for gender differences in the frequency of corresponding authorship causes most observed gender differences in outcomes (panel c) and the gender difference in peer‐review scores ^2^ (panel d) to become nonsignificant. Error bars are present but often smaller than the points. Logistic regression: *PositiveOutcome*[yes/no] = *Journal* + *Year *+ *FirstAuthorGender* + *FirstIsCorrespondingAuthor *+ 2*‐way interactions*; *sent for review*: First is corresponding author: $\chi_{1}^{2}$ = 93.8, *p* < 0.001; first author gender: $\chi_{1}^{2}$ = 0.81, *p* = 0.37; *revision invited (if reviewed)*: First is corresponding author: $\chi_{1}^{2}$ = 5.05, *p* = 0.02; first author gender: $\chi_{1}^{2}$ = 4.57, *p* = 0.03; *revision or resubmission invited (if reviewed)*: First is corresponding author: $\chi_{1}^{2}$ = 11.0, *p* < 0.001; first author gender: $\chi_{1}^{2}$ = 0.93, *p* = 0.33; *revision invited (cumulative, all papers)*: First is corresponding author: $\chi_{1}^{2}$ = 34.0, *p* < 0.001; first author gender: $\chi_{1}^{2}$ = 3.31, *p* = 0.07; *revision or resubmission invited (cumulative, all papers)*: First is corresponding author: $\chi_{1}^{2}$ = 62.3, *p* < 0.001; first author gender: $\chi_{1}^{2}$ = 0.37, *p* = 0.54. Analysis of variance: *ReviewScore* = *Journal* + *FirstAuthorGender* + *FirstIsCorrespondingAuthor* **+ *interaction*. First is corresponding author: *F*_1,11546_ = 9.91, *p* = 0.002; first author gender: *F*_1,11546_ = 0.13, *p* = 0.72.

Papers for which the first author served as corresponding author were 18% more likely to be sent for peer review than were papers for which the first author deferred corresponding authorship to a coauthor (Figure 5a; 59.1 ± 0.4 vs. 50.1 ± 0.8% of papers sent for review). Of papers sent for review, those for which the first author was the correspondent generally received higher peer‐review scores (0.08 standard deviations greater) than those for which the first author deferred corresponding authorship (Figure 5b). Of papers that were reviewed, those for which the first author was corresponding author were substantially more likely to have a positive outcome after review; 9.6% (relative) more likely to be invited for revision and 9.7% likely to be invited for revision or resubmission (Figure 5a). Cumulative across the whole process (including both pre‐ and postreview editorial decisions), papers for which the first author served as corresponding author were 30.1% more likely to be invited for revision (17.3 ± 0.3 vs. 13.3 ± 0.6% positive outcomes) and 30.0% more likely to be invited for revision or resubmission (28.2 ± 0.4 vs. 21.7 ± 0.7% positive outcomes; Figure 5a).

To test whether the higher frequency at which female first authors defer corresponding authorship might contribute to the observed gender differences in peer‐review scores and outcomes, we included corresponding authorship (*FirstIsCorresponding*[yes/no]) in our statistical models testing for effects of gender on peer‐review outcomes. We find that papers with female first authors were just as likely to be sent for peer review (female:male success ratio = 1.02) and obtained similar peer‐review scores if sent for review (mean difference in scores between the genders <0.01 standard deviations; Figure 5c) after accounting for corresponding authorship. We continue to observe a statistically significant gender difference in the probability that reviewed papers have a positive outcome if only invited to revise is considered a positive outcome (Figure 5c; female:male success ratio = 0.92; $\chi_{1}^{2}$ = 4.6, *p* = 0.03), but not if invitation to resubmit is considered also a positive outcome (success ratio = 0.97; $\chi_{1}^{2}$ = 0.93, *p* = 0.33). Cumulative through the entire editorial process (from submission to final decision), papers with female first authors did not differ statistically in their probability of a positive outcome, regardless of whether we consider only an invitation to revise to be a positive outcome (female:male success ratio = 0.93; $\chi_{1}^{2}$ = 3.31, *p* = 0.07) or consider both an invitation to revise and an invitation to resubmit to be positive outcomes (success ratio = 0.98; $\chi_{1}^{2}$ = 0.37, *p* = 0.54; Figure 5c).

### 3.2 Gender differences in outcomes reported by authors

The analyses presented above examine six journals for which we have detailed peer‐review data on all submissions from 2009 to 2015. In this section, we examine editorial outcomes determined from our survey of ecology authors for papers published in 81 journals. The dataset here is different in two notable ways. First, these data are author‐reported survey results. Second, all of the authors surveyed eventually published the paper about which they were surveyed, although many of the submissions were rejected from at least one journal prior to publication. Because our dataset cannot include papers that were rejected and never published, observed rejection rates for journals necessarily underestimate true rejection rates (Paine & Fox, 2018). However, our interest is in gender differences in reported rejection rates, which should be unaffected by these sampling constraints unless male and female authors differ in whether they resubmit and eventually publish previously rejected papers.

**Figure 6:**
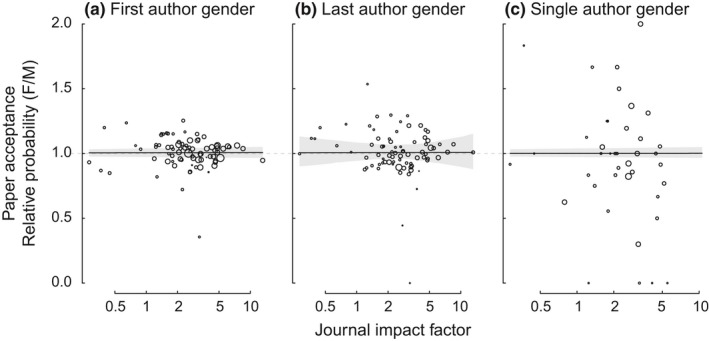
The probability of manuscript acceptance was independent of the gender of (a) the first author, (b) the last author, and (c) the author of single‐authored papers. The success ratio was also independent of journal impact factor. Values >1 indicate that papers with female authors are more likely to have positive outcomes, whereas values <1 indicate that papers with female authors are less likely to have positive outcomes. Logistic mixed‐effect models: *PositiveOutcome[yes/no]* = *JIF* + *AuthorGender*, random effect = *Journal*. JIF X AuthorGender interactions were not significant and were dropped from the models. First author gender (Panel a): JIF: $\chi_{1}^{2}$ = 33.5, *p* < 0.0001, gender: $\chi_{1}^{2}$ = 0.13, *p* = 0.71. Last author gender: (Panel b): JIF: $\chi_{1}^{2}$ = 35.1, *p* < 0.0001, gender: $\chi_{1}^{2}$ = 0.13, *p* = 0.72. Single author gender: (Panel c): JIF: $\chi_{1}^{2}$ = 6.49, *p* = 0.0108, gender: $\chi_{1}^{2}$ = 0.0002, *p* = 0.99.

For papers with multiple authors, we tested whether the gender of the first or last author and journal prominence (journal impact factor) predicted the probability of manuscript acceptance using generalized mixed‐effect models with binomial errors. The probability of a manuscript being accepted for publication from a journal is strongly negatively associated with the impact factor of the journal to which it was submitted (*p* < 0.0001). The gender of the first and senior authors had no detectable effect on the probability of acceptance averaged across all journals (Figure 6 A and B, respectively). There was also no evidence for an interaction between the gender of the first or senior author and journal impact factor on the probability of rejection (*p = *0.90 and *p* = 0.74, respectively). Of papers with just a single author, those with a female author were no more to be accepted than were papers with a male author, regardless of the impact factor of the journal (*p* = 0.98; Figure 6c). We assessed whether the number of journals attempted prior to the acceptance and publication of a study differed between female and male authors using generalized linear models with Poisson‐distributed errors. Papers with male first, last, and sole authors tended to be submitted to more journals prior to acceptance than papers with female authors, but these differences were not significant in all three cases (*p* ≥ 0.52).

### 3.3 Author gender and citations

**Figure 7:**
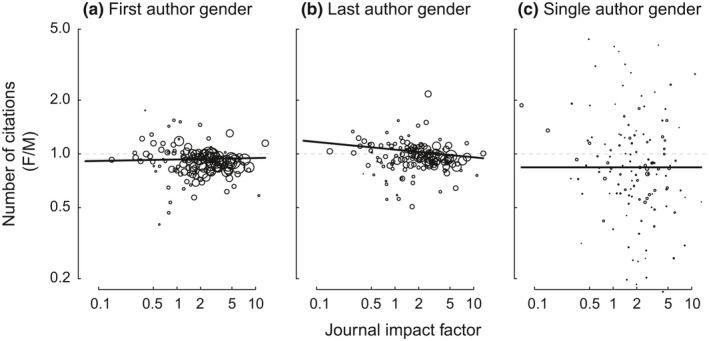
Papers with female first (panel a) or single (panel c) authors were cited less often than papers with male authors irrespective of journal impact factor, whereas the difference in citations obtained by papers with male and female last (senior) authors (panel b) varied with journal impact factor (JIF). Points are the relative number of citations obtained for papers with female versus male authors (female:male ratio), with one point per journal, sized proportional to the number of papers. Solid lines are derived from generalized linear mixed‐effect models, with 95% confidence intervals estimated through parametric bootstrapping. Papers with female first authors were cited slightly less than papers with male first authors, though this disparity lessened at high JIF journals. Papers with male last authors were to be better cited than papers with female last authors at highly prominent journals, whereas papers with female senior authors tended to be more cited in less prominent journals. Papers with female sole authors were consistently cited less well than papers with sole male authors. Values >1 indicate that papers with female authors had positive outcomes, whereas values <1 indicate that papers with female authors had negative outcomes. Generalized linear mixed‐effect models with Poisson errors: *NumberCitations = JIF *+* AuthorGender *+ Interaction, random effect = *PublishingYear*. First author gender (Panel a): JIF: $\chi_{1}^{2}$ = 367,763, *p* < 0.0001, gender: $\chi_{1}^{2}$ = 1,194, *p* < 0.0001, JIF × gender: $\chi_{1}^{2}$ = 7.069, *p* = 0.008. Last author gender: (Panel b): JIF: $\chi_{1}^{2}$ = 373,698, *p* < 0.0001, gender: $\chi_{1}^{2}$ = 1.24, *p* = 0.27, JIF x gender: $\chi_{1}^{2}$ = 151.0, *p* < 0.0001. Single author gender: (Panel c): JIF: $\chi_{1}^{2}$ = 25,727, *p* < 0.0001, gender: $\chi_{1}^{2}$ = 351.2, *p* < 0.0001, JIF × gender: $\chi_{1}^{2}$ = 0.468, *p* = 0.49.

**Figure A5:**
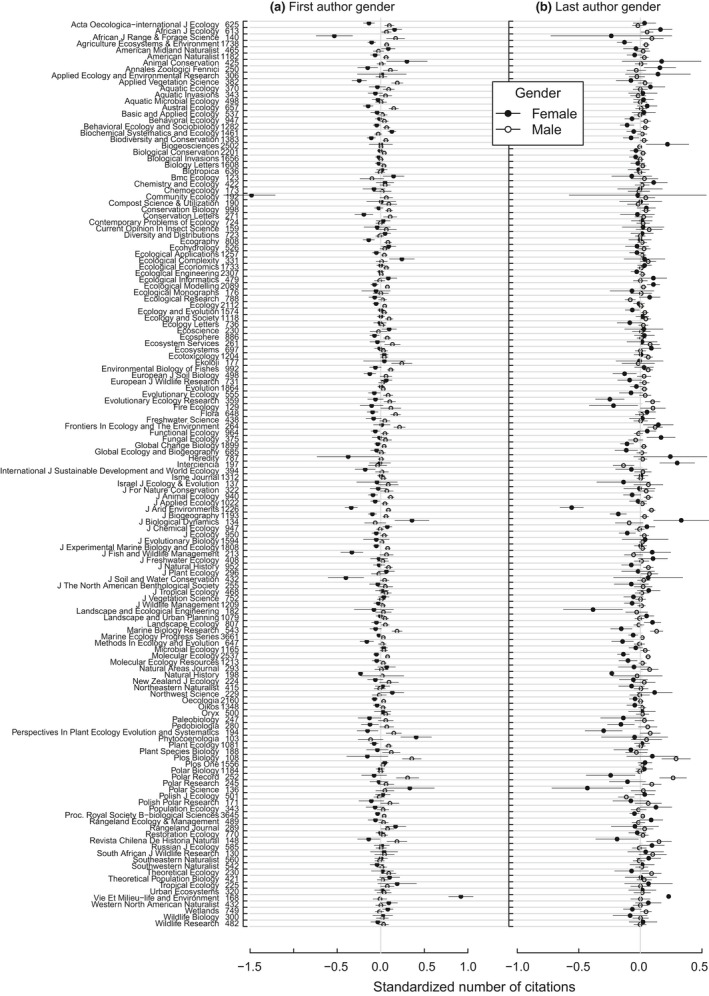
Number of citations gained per paper, as predicted by journal and gender of the (a) first author or (b) last author. The log_10_ transformed number of citations is standardized to mean 0 and standard deviation of 1 within each journal. Plotted are means ± *SEM*. Sample sizes per journal are listed beside the journal name

For papers with multiple authors, the number of citations obtained by a published article varied significantly among journals, with higher impact factor journals obtaining more citations (Appendix Figure A5; Figure 7). We thus included journal impact factor (JIF) as a covariate in our analyses, so that we are asking whether papers with female first authors are cited differently than papers with male first authors within journals of the same average impact.

Papers with male first authors were slightly but consistently better cited than were papers with female first authors (Figure 7a). The effect diminished from 8% to 4% in journals of greater impact factor (gender‐x‐JIF interaction; $\chi_{1}^{2}$ = 7.07, *p* = 0.008). The pattern for senior author gender differed, in that papers with male last authors were better cited than papers with female last authors, but only at high JIF journals (Figure 7b). At lower‐JIF journals, the opposite pattern was observed; papers with female senior authors were better cited (gender‐x‐JIF interaction, $\chi_{1}^{2}$ = 151.0, *p* < 0.0001). For example, at a journal of JIF 10, papers with male senior authors gained 4.7% more citations than those with female senior authors, whereas at a journal with JIF of 0.1, papers with female senior authors obtained 7.4% more citations than those with male senior authors. Because the number of citations obtained by papers published by journals of JIF 0.1 versus JIF 10 varies by two orders of magnitude, this difference compounds to give male senior authors a dramatic advantage in the accumulation of citations. For papers with just a single author, those with a single female author received, on average, 84% as many citations as did papers with a single male ($\chi_{1}^{2}$ = 351.2, *p* < 0.0001; Figure 7c).

**Figure 8:**
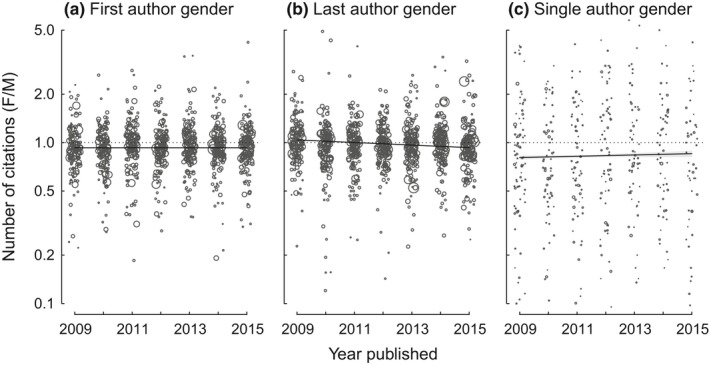
Variation through time in the number of citations obtained by articles varied with gender of the (a) first author, (b) last author, and (c) sole author. Plotted are predictions from a mixed‐effect model of year * gender, with journal as a random effect. Points represent the ratio of the citations gained by papers published by female and male authors in journal by year combinations and are sized proportional to the number of papers assessed. For the last author model only, the interaction between year and gender was significant. Thus, from 2009 to 2015, papers with male authors generally but not consistently obtained more citations than did papers with female authors. Values >1 indicate that papers with female authors obtain more citations, whereas values <1 indicate that papers with female authors obtain fewer citations. Generalized linear mixed‐effect models: *NumberCitations* = *PublishingYear* + *AuthorGender*, random effect = *Journal*. First author gender (Panel a): year: $\chi_{1}^{2}$ = 302,720, *p* < 0.0001, gender: $\chi_{1}^{2}$ = 1633, *p* = < 0.0001, year × gender: $\chi_{1}^{2}$ = 1.88, *p* = 0.17. Last author gender: (Panel b): year: $\chi_{1}^{2}$ = 307,265, *p* < 0.0001, gender: $\chi_{1}^{2}$ = 3.66, *p* = 0.055, year × gender: $\chi_{1}^{2}$ = 284.7, *p* < 0.0001. Single author gender: (Panel c): year: $\chi_{1}^{2}$ = 14,245, *p* < 0.0001, gender: $\chi_{1}^{2}$ = 432.7, *p < *0.0001, year x gender: $\chi_{1}^{2}$ = 3.16, *p* = 0.075.

Over the timeframe of this study (2009–2015), gender differences in the number of citations gained by papers with female and male first or single authors remained largely consistent; papers with male authors consistently obtained more citations than did papers with female authors (Figure 8a,c). For male and female last authors, however, the number of citations shifted from slightly favoring female authors in 2009 to slightly favoring male authors in later years (gender‐x‐year interaction; $\chi_{1}^{2}$ = 284.7, *p* < 0.0001; Figure 8b).

## 4 DISCUSSION

Controlled experiments commonly find that the performance of women is scored more negatively than that of men when no actual difference exists. However, the extent to which such gender biases influence editors and peer reviewers remains uncertain. Despite a few high‐profile examples, most studies find no gender difference in the outcomes of peer review at academic journals, though there are some notable exceptions. In our study, we find that papers with female first authors are equally likely to be sent for peer review as are papers with male first authors, but they obtain slightly lower peer‐review scores and are less likely to have a positive outcome after peer review, though the magnitude of this gender difference varied among journals. The gender differences in both peer‐review scores and editorial decisions appear to be partly due to gender differences in authorial roles. Papers for the which the first author deferred corresponding authorship to a coauthor obtained (on average) substantially lower peer‐review scores and were less likely to have positive outcomes. Gender differences in corresponding authorship explained some of the gender differences in peer‐review scores and the frequency of positive editorial decisions. After publication, we also find that published papers with female first, last, or single authors are cited less often than those with male authors.

### 4.1 Gender differences in peer‐review outcomes

Our analyses uncover differences in editorial and peer‐review outcomes between papers authored by men and those authored by women. Though many of our individual analyses found no significant gender differences, the effects are consistently in the same direction: Papers with female authors obtain lower peer‐review scores and have lower probabilities of positive editorial decisions, than do papers with male authors. Effect sizes varied throughout stages of the process and across journals but, cumulative from submission through to the editorial decision, papers with female authors were, on average, 4 to 9% less likely (depending on author position; Table 1) to be invited for revision and/or resubmission than were papers with male authors (female:male success ratios of 0.96 to 0.91, averaged across journals and years).

**Table 1:** The cumulative disparity in relative success rates for papers authored by women compared to men

	Relative probability of positive outcome cumulative through entire review process Female/Male authors	
	Revision invited	Revision or resubmission invited
First author	0.925 ± 0.045	0.958 ± 0.020
Senior author	0.948 ± 0.033	0.905 ± 0.026
Corresponding author	0.914 ± 0.039	0.963 ± 0.026

Values are the probability of a positive outcome (female author)/probability of a positive outcome (male author), cumulative through the entire editorial and peer‐review process. Values <1 indicate that papers with female authors are less likely to have a positive outcome. Means are averaged across journals, with the reported standard error calculated from the among‐journal variance.

Our conclusion, that papers authored by women are less likely to have positive outcomes, contrasts with the conclusions of many previous studies of peer review at academic journals, albeit with some exceptions (summarized in the Introduction, above). Though most studies conclude that men and women have equal success rates at journals, many of these studies observe trends toward papers with male authors being more likely to be accepted for publication (e.g., 7%–12% more likely in Heckenberg & Druml, 2010; Primack et al., 2009), as reported here. Indeed, a previous study of the journal *Functional Ecology* (Fox et al., 2016), one of the journals included in the current study, observed trends similar to those reported here, though none were statistically significant.

We draw two important conclusions from this variation in conclusions among research studies. First, the presence of gender differences and magnitude of effects almost certainly vary among disciplines and journals. Second, very large sample sizes are necessary to detect small but meaningful (e.g., 5%–10%) gender differences in peer‐review outcomes. This is because of the tremendous amount of background variation due to heterogeneity in manuscript quality and in editor and reviewer populations. The large sample size of the current study, >23,000 papers submitted to six journals, provides the statistical power necessary to detect gender differences in the range of 5%–10%. It is notable that the previous studies that have provided the most compelling evidence of gender differences in peer review are of similarly large scale. For example, of >23,000 papers submitted to *eLife*, those authored by women were ~12% less likely to be accepted for publication than those authored by men (Murray et al., 2018). Similarly, of >8,500 manuscripts submitted to three *Frontiers* journals (Walker et al., 2015), papers authored by women obtained lower peer‐review scores than papers authored by men. However, at least one large study found the opposite; in an analysis of >22,000 papers submitted to journals of the American Geophysical Union, Lerback and Hanson (2017) found that papers authored by women had ~7% *higher *acceptance rates. It is thus clear that gender discrepancies vary a lot among journals, both within and among studies, and that large sample sizes are necessary to detect these differences when they exist.

What explains the discrepancy in success rates between men and women in our study? One possibility is that reviewers and/or editors discriminate against papers by female authors during their assessments of manuscript quality, novelty, or significance. Biases in which the performance or products of women are evaluated less positively than that of men have been demonstrated in a wide variety of contexts (discussed above). Unfortunately, our data do not allow us to directly test for unconscious or conscious biases because we have no independent metrics of manuscript quality and significance. Explanations other than gender discrimination could contribute to the gender disparities observed here. For example, women defer submission of their manuscripts to collaborators more often than do men and might use different criteria for evaluating the journals to which they send their papers (Regazzi & Aytac, 2008), such that submitted papers are, on average, slightly different between male and female authors. Though we cannot test these hypotheses, the importance of considering alternatives to gender discrimination is highlighted by Ledin et al. (2007). They observed that gender differences in success rate at obtaining fellowships from the European Molecular Biology Organization persisted when committees were blinded to applicant gender. Though not directly comparable to our study, in part because fellowship applications are reviewing applicant productivity rather than manuscript quality, the results of Ledin et al. (2007) highlight that gender differences in success rates can arise from factors other than discrimination (but see Witteman, Hendricks, Straus, & Tannenbaum, 2019 for a counterexample). Our results are highly suggestive of a problem, but hypotheses to explain the gender discrepancies observed here can only be tested with controlled experiments. In particular, we argue that a controlled experiment in which real journal submissions are randomly assigned to blind versus nonblind peer review, should be performed by one or more ecology journals, to test for gender discrimination (and other potential biases) in editorial and peer review. Such an experiment has recently been announced by the journal *Functional Ecology* (one of the journals considered in our study)(Fox et al., 2019). Similar experiments have been performed by nonecological journals, but few (Blank, 1991; Carlsson et al., 2012; Ross et al., 2006) have tested for evidence of gender discrimination in nonblinded manuscripts.

One striking result of our analyses is that papers for which the first author is also the corresponding author perform much better throughout all stages of the manuscript review process. Such papers were 18% more likely to be sent for peer review, obtained higher scores from reviewers, and were 10% more likely to be invited for revision or resubmission after review, with a cumulative 30% higher probability of a positive outcome across the entire review process. This is a strikingly large effect that warrants further investigation. We think it unlikely that biases against authors who defer corresponding authorship can explain an effect this large. Instead, we suspect the low success of papers being corresponded by someone other than the first author is because either: (a) These papers are being written, at least in part, by someone less familiar with (or less committed to) the research being described in the manuscript, such as a research mentor or a colleague more fluent in English; or (b) first authors are more willing to defer corresponding authorship when a paper is of lower significance and/or reports less robust research. Regardless of the explanation, this difference may be important for understanding gender differences in publishing success because women defer corresponding authorship more often than do men (Edwards et al. 2018; Fox et al., 2018), possibly because they are more likely than men to leave science (Adamo, 2013). Our results suggest that the gender difference in corresponding authorship contributes to the gender difference in peer‐review outcomes; including corresponding authorship in our statistical models causes first author gender differences to become statistically nonsignificant (cumulative through the entire process). However, the degree to which considering corresponding authorship changes estimated female:male success ratios is small, suggesting that gender differences in corresponding authorship, although possibly a contributing factor, are not enough to account for all of the observed gender differences in peer‐review outcomes.

Our analysis of peer‐review outcomes is limited to just six journals for which we have detailed data on all submissions. To better understand potential gender biases across the entire ecology literature, we tested for gender differences in a dataset collected via an author survey of manuscript publication histories. In this survey, we asked authors to provide the complete submission history for their published paper—to which journal each manuscript had previously been submitted and the outcomes of each separate submission. Interestingly, we observe no evidence a gender difference in the author‐reported outcomes of editorial review in this survey data; papers by female authors were no more likely to report having been rejected by one or more journals before eventual publication. The effect size, averaged across journals, was very close to 0 (Figure 6), with the sign of the effect opposite that in our peer‐review dataset; that is, not even suggestive of bias against papers authored by women. One possible explanation for the difference in conclusion between these two datasets is that papers about which we survey authors include only the subset of papers that are eventually published, and thus represent a biased sample of all papers that are reviewed; papers that are rejected from one journal and never published anywhere are unknown to us, and thus not included in our sample. If women are less likely than men to resubmit their paper (to another journal) following rejection, the rejection rate observed in our survey data could be biased against detecting rejections of papers with female authors. Some evidence suggests that women respond differently to social and peer rejection than do men (Stroud, Salovey, & Epel, 2002; Vanderhasselt, Raedt, Nasso, Puttevils, & Mueller, 2018), though it is unclear if this occurs in the academic publishing context. Also, because women leave science more often than do men (Adamo, 2013), they may not be able or willing to resubmit papers following rejection. Alternatively, men and women may respond differently to the survey itself. Estimated rejection rates from survey responses underestimate rejection rates obtained directly from individual journals (Paine & Fox, 2018). This suggests that our survey is either missing a population of papers that were submitted, rejected, and never eventually published, or that authors who had more positive experiences with their manuscripts are more likely to reply (survey response rates were higher for papers with male first authors, 21.3% vs. 17.5% for male vs. female authors). Or, possibly, the difference in conclusions reached from these two datasets (our submitted papers dataset including just six journals vs. the survey dataset including all ecology journals) may indicate that gender difference observed at these six journals does not extend to the ecology literature more broadly, though we think this unlikely.

### 4.2 Gender difference in citations obtained

We find that papers with female first authors are ~2% less well cited, on average, than papers with male first authors, even after correcting for the impact factor (a metric of average citation rates) of the publishing journal. Papers with female last authors are also less well cited in higher impact factor journals, though the reverse is true in low impact factor journals (in which total citations obtained are very low), with the cumulative effect being that papers with female authors receive fewer citations than papers with male authors. This observation contrasts with a number of previous studies of the ecology literature, which found that citation rates did not differ between men and women (Borsuk et al., 2009; Cameron et al., 2016; Leimu & Koricheva, 2005). However, our result is consistent with results in a variety of other fields (e.g., Bendels et al., 2018 and references therein), many of which find that papers with female first and/or senior authors are cited ~1.5%–8% less often than are papers with male authors, a range that encompasses the effect sizes measured in our study.

One explanation for the observed gender difference in citations could be that men self‐cite more often than do women, something observed in a variety of fields, including ecology (Cameron et al., 2016). Men tend to publish more often than do women and thus collaborate with more distinct coauthors over their careers than do women (Zeng et al., 2016), such that they should benefit more than do women from both self‐citation and citation by their collaborators (Leblond, 2012). Unsurprisingly, self‐citation inflates average citations obtained per paper and inflates metrics of career‐long impact, such as the h‐index (Cameron et al., 2016; Engqvist & Frommen, 2008). Unfortunately, we do not have self‐citation data in our dataset, so cannot test the hypothesis that self‐citation and/or citation by collaborators accounts for our observed gender differences in citations obtained.

## 5 CONCLUSIONS

Many studies have demonstrated that the performance of women is generally rated lower than that of men, even in controlled experiments. However, the degree to which this bias impacts the editorial and peer‐review processes that underlie academic publishing has been controversial. Some studies of submitted grants and manuscripts find discrepancies in peer‐review scores or final outcomes between male and female authors, but most do not. In our study of >23,000 manuscripts submitted to six journals of ecology and evolution, we find evidence that papers authored by women receive lower peer‐review scores and, cumulative across the entire manuscript review process, are ~4%–10% (depending on author position) less likely to be invited for revision or resubmission (Table 1). We believe that our data make a compelling case for there being a meaningful discrepancy in outcomes between papers authored by men and women. However, we caution that our data do not allow us to test hypotheses regarding causes of this discrepancy. Though our data are consistent with predictions of the gender differences we would observe if editors and/or reviewers discriminate against women, other causes may contribute to explaining the difference. For example, women defer submission of manuscripts to coauthors more often than do men (possibly because women leave science more often than do men), and our data show that deferring corresponding authorship to collaborators is a significant predictor of whether manuscripts fare well during peer review. Women may also make different decisions regarding choice of journal or may respond differently to prior rejection (from a different journal) leading to differences in the manuscripts submitted to our study journals. We have no data to test these explanations, but argue that these and other hypotheses must be explored further—such as by performing a controlled randomized experiment on submissions to a high‐profile ecology journal—if we are to understand whether and how much discrimination in the editorial process influences publishing in the scholarly literature. Regardless of the underlying causes of the gender differences observed here, our results are consistent with the widespread perception that academic publishing discriminates against women. We thus argue that journals should consider changes to peer‐review procedures to address this perception, and possible reality, of gender discrimination, such as mandatory double‐blind peer review, or limiting author given names to first and middle initials (which do not signal gender unless the author is already known by surname to the reviewer) until the completion of peer review.

## CONFLICT OF INTEREST

The authors have no conflict of interest.

## AUTHOR CONTRIBUTIONS

CWF collected and analyzed the data for the sections of the manuscript examining papers submitted to *Evolution* and the five journals of *British Ecological Society*. CETP collected and analyzed the data for the author survey and manuscript citations. Both CWF and CETP wrote the manuscript.

## Figures and Tables

**Figure A2:**
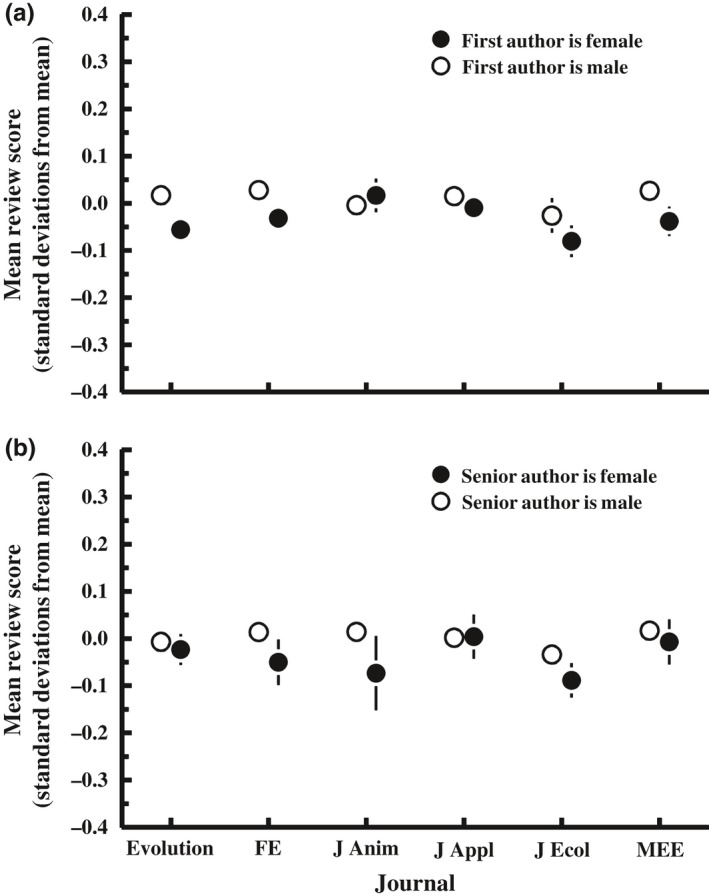
The scores given to papers by reviewers. Review scores were standardized to a mean of 0 and a standard deviation of 1.0 within journals and years so that all journals are on the same scale. Means (±*SE*) are averages across years within journals. Analyses as in Figure 2

**Figure A3:**
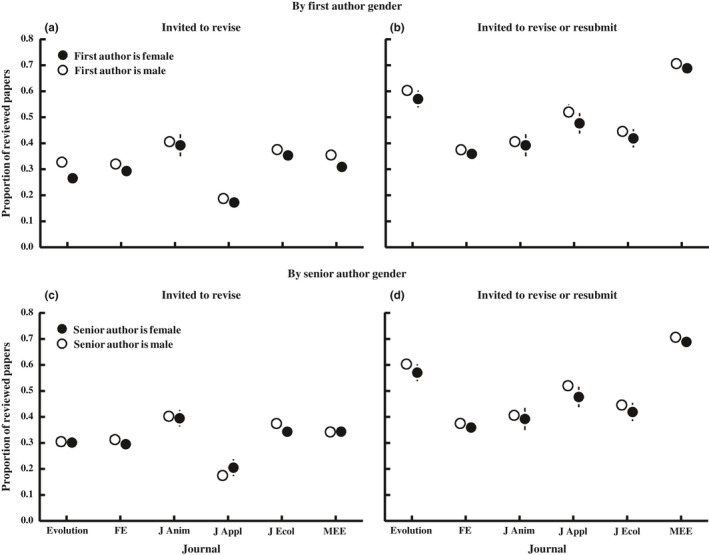
The proportion of reviewed papers that had positive final outcomes. The left panels count as a positive outcome‐only papers that were invited for minor or major revision. The right panels include papers that were invited either for minor or major revision or that were rejected but invited to resubmit. Three of the journals make frequent use of “reject with resubmission invited,” but three others use this decision category rarely. Analyses as in Figure 3

**Figure A4:**
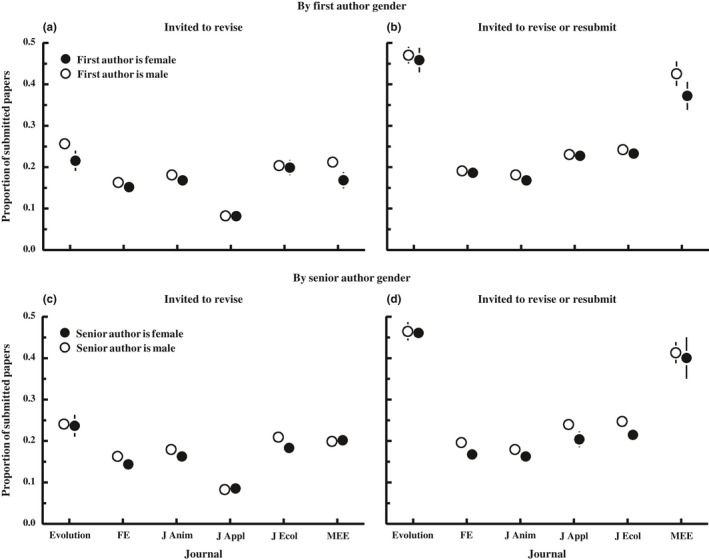
The proportion of all submitted papers that had positive final outcomes, cumulative through the entire prereview and postreview process. This figure differs from Figure 3 in that negative outcomes include both papers that were declined without review (desk rejections) and those declined after review. The left panels count as a positive outcome‐only papers that were invited for minor or major revision. The right panels include papers that were invited either for revision or were rejected but invited to resubmit. Analyses as in Figure 4

## Data Availability

An anonymized version of the submitted papers dataset, removing personal identifying information (manuscript titles, author names, and author countries), is available on Dryad, https://doi.org/10.5061/dryad.7p048mk. An anonymized version of the author questionnaire dataset is also available on Dryad (https://doi.org/10.5061/dryad.6nh4fc2).

## APPENDIX 1

### ADDITIONAL ANALYSES

#### Author gender ratio

In this section, we consider author gender ratio (proportion of authors that are women = number of women/total number of authors) as our independent variable. The author gender ratio did not predict the probability that a manuscript was sent for peer review (logistic regression, statistical model as in Figure 1, except that *AuthorGender *is a covariate rather than categorical variable; $\chi_{1}^{2}$ = 3.34, *p* = 0.07). However, consistent with previous analyses, of papers sent for review, those with a higher proportion of female authors obtained lower average peer review scores (*F*_1,11757_ = 8.46, *p* = 0.004), and were less likely to have a positive decision after review whether we consider only an invitation to revise ($\chi_{1}^{2}$ = 9.39, *p* = 0.002) or an invitation to revise or resubmit ($\chi_{1}^{2}$ = 11.3, *p* < 0.001) as positive outcomes. However, this gender difference disappeared once accounting for the difference in peer‐review scores; after taking account of the lower scores received by papers with more female authors (models in Figure 3, with author gender ratio added as a covariate), the effect of author gender ratio was nonsignificant for both models, including only revision invitations ($\chi_{1}^{2}$ = 0.88, *p* = 0.34) or both revision and resubmission invitations ($\chi_{1}^{2}$ = 1.76, *p* = 0.18) as positive outcomes.

Cumulative through the entire process (pre‐ and postreview editorial decisions), papers with a higher proportion of female authors were less likely to have a positive outcome, whether invited to submit a revision ($\chi_{1}^{2}$ = 13.4, *p* < 0.001) or invited to revise +resubmit ($\chi_{1}^{2}$ = 14.8, *p* < 0.001).
